# A Novel Cell Line Based Orthotopic Xenograft Mouse Model That Recapitulates Human Hepatoblastoma

**DOI:** 10.1038/s41598-017-17665-8

**Published:** 2017-12-19

**Authors:** Sarah E. Woodfield, Yan Shi, Roma H. Patel, Jingling Jin, Angela Major, Stephen F. Sarabia, Zbigniew Starosolski, Barry Zorman, Siddharth S. Gupta, Zhenghu Chen, Aryana M. Ibarra, Karl-Dimiter Bissig, Ketan B. Ghaghada, Pavel Sumazin, Dolores López-Terrada, Sanjeev A. Vasudevan

**Affiliations:** 1 Divisions of Pediatric Surgery and Surgical Research, Michael E. DeBakey Department of Surgery, Texas Children’s Surgical Oncology Program, Texas Children’s Liver Tumor Program, Dan L. Duncan Cancer Center, Baylor College of Medicine, Houston, TX 77030 USA; 2 Department of Pediatrics, Dan L. Duncan Cancer Center, Baylor College of Medicine, Houston, TX 77030 USA; 3 Department of Pathology and Immunology, Dan L. Duncan Cancer Center, Baylor College of Medicine, Houston, TX 77030 USA; 4 Singleton Department of Pediatric Radiology, Texas Children’s Hospital, Houston, TX 77030 USA; 5 Center for Cell and Gene Therapy, Stem Cells and Regenerative Medicine Center, Department of Molecular and Cellular Biology, Dan L. Duncan Cancer Center, Graduate Program Department of Molecular and Cellular Biology, Program in Developmental Biology, and Program in Translational Biology and Molecular Medicine, Baylor College of Medicine, Houston, TX 77030 USA

## Abstract

Currently, preclinical testing of therapies for hepatoblastoma (HB) is limited to subcutaneous and intrasplenic xenograft models that do not recapitulate the hepatic tumors seen in patients. We hypothesized that injection of HB cell lines into the livers of mice would result in liver tumors that resemble their clinical counterparts. HepG2 and Huh-6 HB cell lines were injected, and tumor growth was monitored with bioluminescence imaging (BLI) and magnetic resonance imaging (MRI). Levels of human α-fetoprotein (AFP) were monitored in the serum of animals. Immunohistochemical and gene expression analyses were also completed on xenograft tumor samples. BLI signal indicative of tumor growth was seen in 55% of HepG2- and Huh-6-injected animals after a period of four to seven weeks. Increased AFP levels correlated with tumor growth. MRI showed large intrahepatic tumors with active neovascularization. HepG2 and Huh-6 xenografts showed expression of β-catenin, AFP, and Glypican-3 (GPC3). HepG2 samples displayed a consistent gene expression profile most similar to human HB tumors. Intrahepatic injection of HB cell lines leads to liver tumors in mice with growth patterns and biologic, histologic, and genetic features similar to human HB tumors. This orthotopic xenograft mouse model will enable clinically relevant testing of novel agents for HB.

## Introduction

Hepatoblastoma (HB) is the most common malignant liver tumor seen in children^1^. The disease is most often diagnosed in patients under five years of age and is usually sporadic but can also be associated with familial adenomatous polyposis, Beckwith-Wiedemann syndrome, or prematurity^2^. Five-year overall survival (OS) of patients with stage I and II disease is above 95%, but patients with stage IV disease have a five-year OS rate of about 40%^3^. Standard treatment for HB consists of surgery and high dose, non-targeted chemotherapy, which leads to multiple damaging and long term side effects, including ototoxicity and cardiotoxicity^4–6^. Thus, new treatment strategies are needed, especially for high-risk patients.

To date, *in vivo* HB research includes studies with hydrodynamic injection of oncogenes for liver specific expression^7^, as well as subcutaneous and intrasplenic murine xenograft models^8–10^. Unfortunately, these models do not recapitulate the disease seen in a majority of patients, which is a large primary tumor encompassing one to four segments of the liver^3^. Mice with tumors generated with hydrodynamic injection develop multifocal nodules within the liver, and the organ is eventually entirely replaced by tumor. This may be representative of patients that present with tumor in all four segments of the liver, but this is only a small percentage of patients^3^. With the subcutaneous and intrasplenic xenograft models, tumors can be quickly generated in genetically identical animals from the human HB cell lines Huh-6^11^, HepT1^8^, and HepG2^12^. In the subcutaneous model, injection of all three cell lines led to growth of tumors, depending on the strain of mice and time elapsed since injection of cells^8,9^. In the intrasplenic model, immunodeficient mice were directly injected with HepG2, Huh-6, or HepT1 cells into the spleen. The Huh-6 and HepT1 tumor cells, but not HepG2 cells, then migrated to the liver, giving rise to intrahepatic tumors^9,10^. Of note, animals that underwent splenectomy just after injection more readily developed intrahepatic tumors^10^. These tumors were small, multifocal nodules that again do not represent the disease typically seen in children. Notably, there is one published study of injection of HepG2 cells into the portal vein to generate intrahepatic tumors, but the focus in this work is use of this model for drug testing for hepatocellular carcinoma (HCC)^13^. Thus, although these models have contributed to the field, none truly recapitulates the disease. For effective preclinical studies to be performed, a true intrahepatic orthotopic xenograft model that accurately replicates the human disease is essential. We have successfully developed an intrahepatic patient-derived xenograft (PDX) model of HB using patient specimens^14^. Other groups have also examined subcutaneous and intrahepatic growth of patient-derived liver cancer tissues as models of HCC, including an interesting study in which tumors composed of sorted human liver cancer stem cells (hLCSCs) were grown subcutaneously^15,16^. Since these tissues have limited availability due to the rarity of the disease, we wanted to develop and characterize an intrahepatic, orthotopic xenograft model using commercially available HB cell lines. In addition, cell line derived xenograft models can be better standardized and are not dependent on tissue quality of surgical samples that usually have been exposed to chemotherapy.

In this paper, we describe the development and characterization of such an intrahepatic xenograft HB mouse model. Human HB cells were injected into the livers of immunocompromised mice. Mice were monitored for tumor growth using bioluminescence imaging (BLI), magnetic resonance imaging (MRI), and measurement of serum levels of human α-fetoprotein (AFP). At the conclusion of the study, animals were euthanized and tissues were harvested for protein expression analysis by immunohistochemistry and gene expression profiling by RNA sequencing.

## Results

### Generation of tumors by intrahepatic injection of human HB cell lines

**Figure 1:**
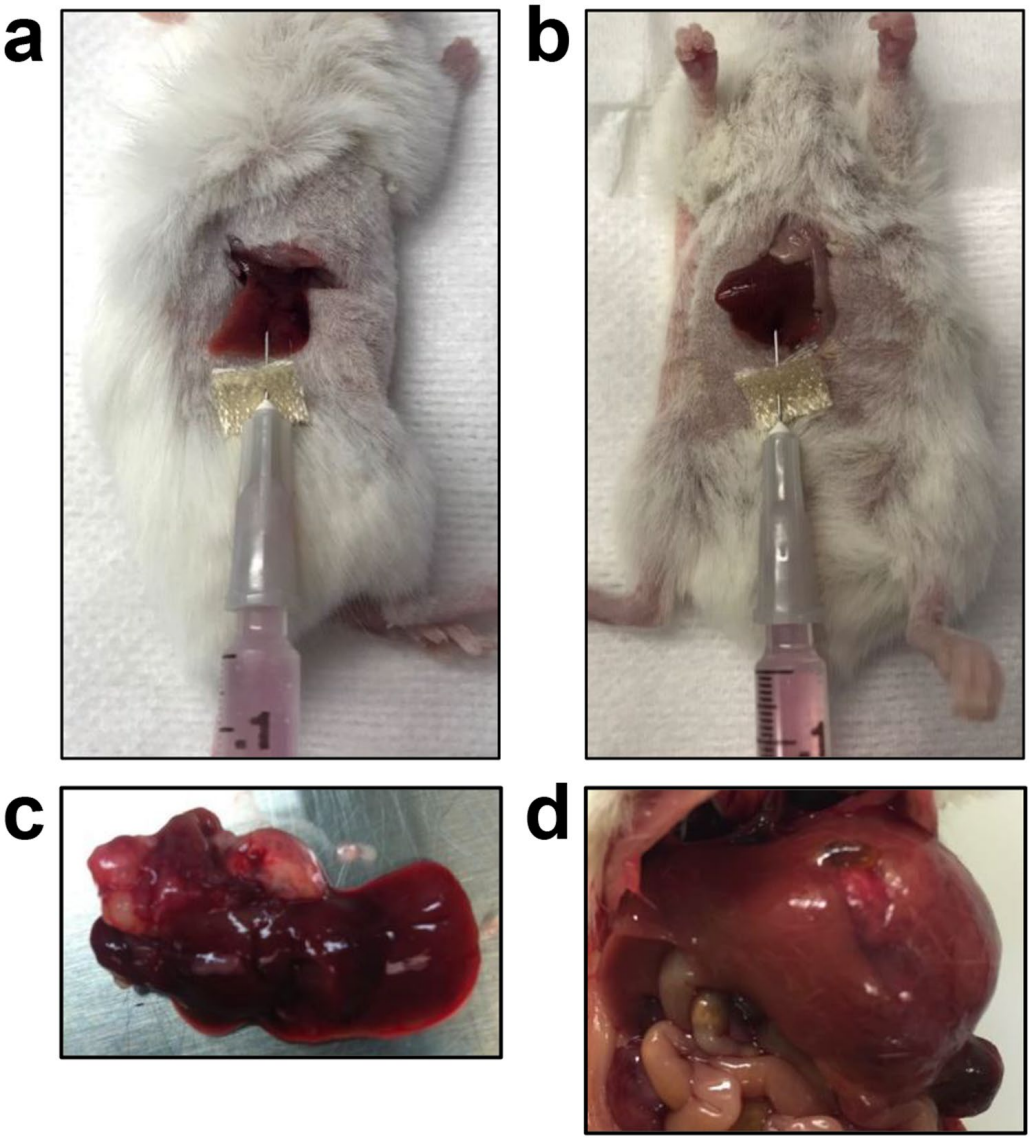
Injection of HB cells into the mouse liver to generate xenograft tumors. Cells were injected either into the right median lobe (**a**) or the left lateral lobe (**b**). (**c**,**d**) Representative gross tumors generated with injection of Huh-6 cells into the right median lobe (**c**) or HepG2 cells into the left lateral lobe (**d**).

To generate liver tumors, we injected two million HepG2 or Huh-6 human HB cell lines into either the right median lobe with a right flank incision (Fig. 1a) or the left lateral lobe with a midline, abdominal incision (Fig. 1b) of the liver of NOD/Shi-*scid*/IL-2Rγ^null^ (NOG) mice (see methods). Regardless of injection technique, the tumors grew as large, exophytic masses originating from the injected lobe and invaded multiple adjacent segments of the liver as seen in children with pre-treatment extent of disease (PRETEXT) I, II, and III tumors (Fig. 1c,d) ^17^.

**Figure 2:**
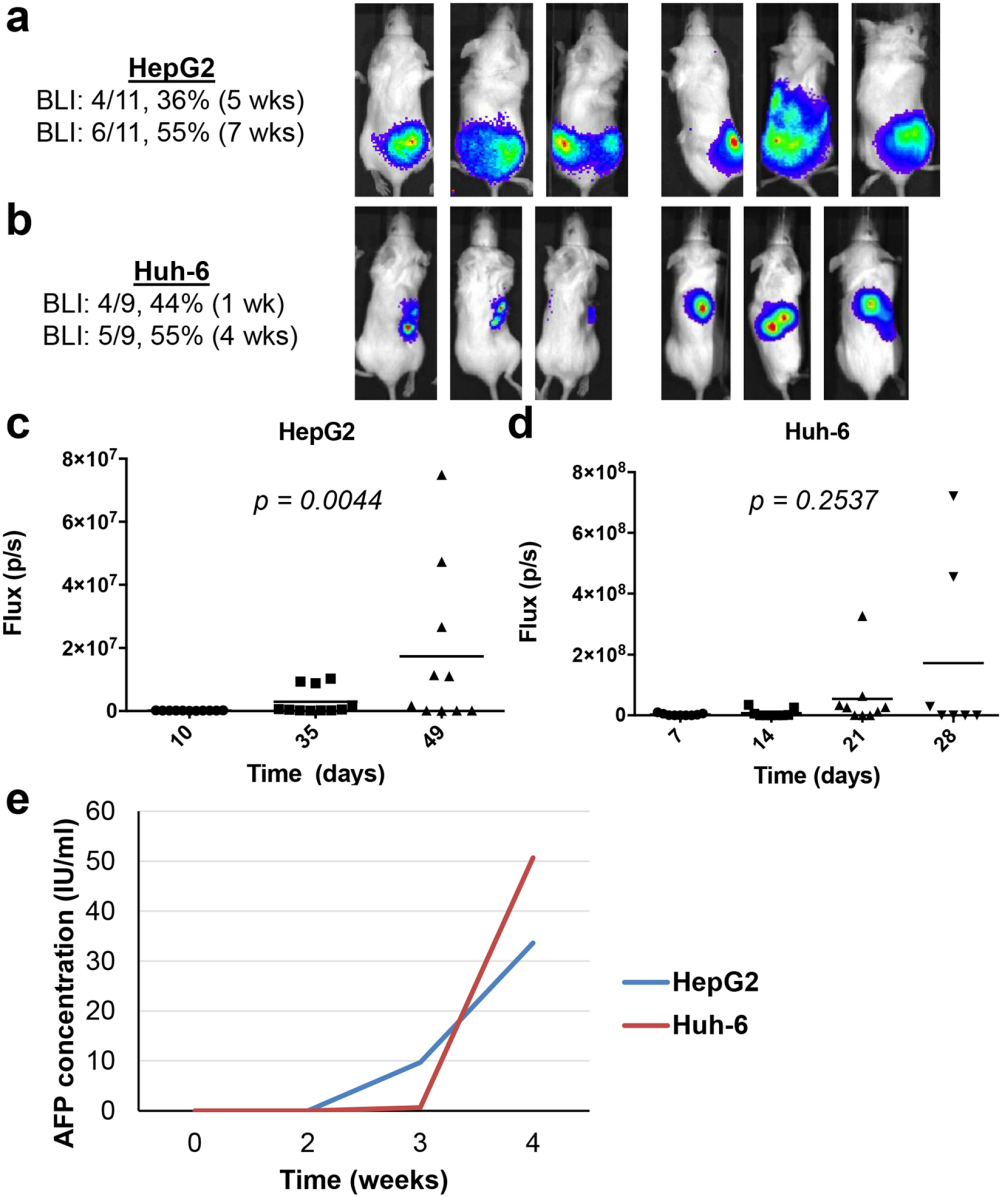
BLI signals and serum AFP levels of mice injected with HB cells. (**a**,**b**) Three representative mice with HepG2 (**a**) or Huh-6 (**b**) xenografted tumors shown at early and late time points with number of mice growing tumors indicated. (**c**,**d**) Increase in BLI signals shown at each time point with associated *p* values determined with the Kruskal-Wallis test to indicate significance. (**e**) Serum human AFP increases with tumor growth in mice harboring xenograft tumors.

In order to longitudinally monitor the growth of tumors *in vivo*, we used cells that had been transduced with lentiviral vectors expressing the *luciferase* gene. The expression of strong luciferase activity (2–3 million relative luminescence (RLU)) was confirmed prior to implantation of tumor cells. Upon intraperitoneal injection of luciferin into the animals, the cells emitted a BLI signal that could be monitored from week to week (Fig. 2a–d). Four of 11 (36%) mice injected with HepG2 cells showed BLI signal at 5 weeks after injection, and 6 of 11 (55%) displayed BLI signal at 7 weeks (Fig. 2a). Four of 9 (44%) mice injected with Huh-6 cells demonstrated BLI signal at 1 week after injection, and 5 of 9 (55%) exhibited BLI signal at 3 weeks after injection (Fig. 2b). Temporal analysis of BLI signals reflects the growth of tumors (Fig. 2c,d) and shows that changes in the HepG2 masses are significant (*p = 0*.*0044*). Taken together, the data demonstrates that intrahepatic injection of both HB cell lines successfully leads to the growth of liver tumors in immunocompromised mice.

### Human AFP is elevated in the serum of mice harboring xenograft tumors

One of the most important indicators used for diagnosis and disease surveillance in patients with HB is levels of AFP in the blood, and 97% of patients show elevated levels^18^. We hypothesized that levels of human AFP would be elevated in the serum of the mice harboring the xenograft tumors. To test this hypothesis, we measured serum human AFP levels weekly in one mouse representative of each cell line xenograft using an enzyme-linked immunosorbent assay (ELISA). At the time of injection of cells and at one week after injection, levels of human AFP in the blood remained very low (Fig. 2e). By three weeks after injection, animals with both xenograft tumors showed elevated human AFP in their serum (Fig. 2e).

### MRI of xenograft tumors

**Figure 3:**
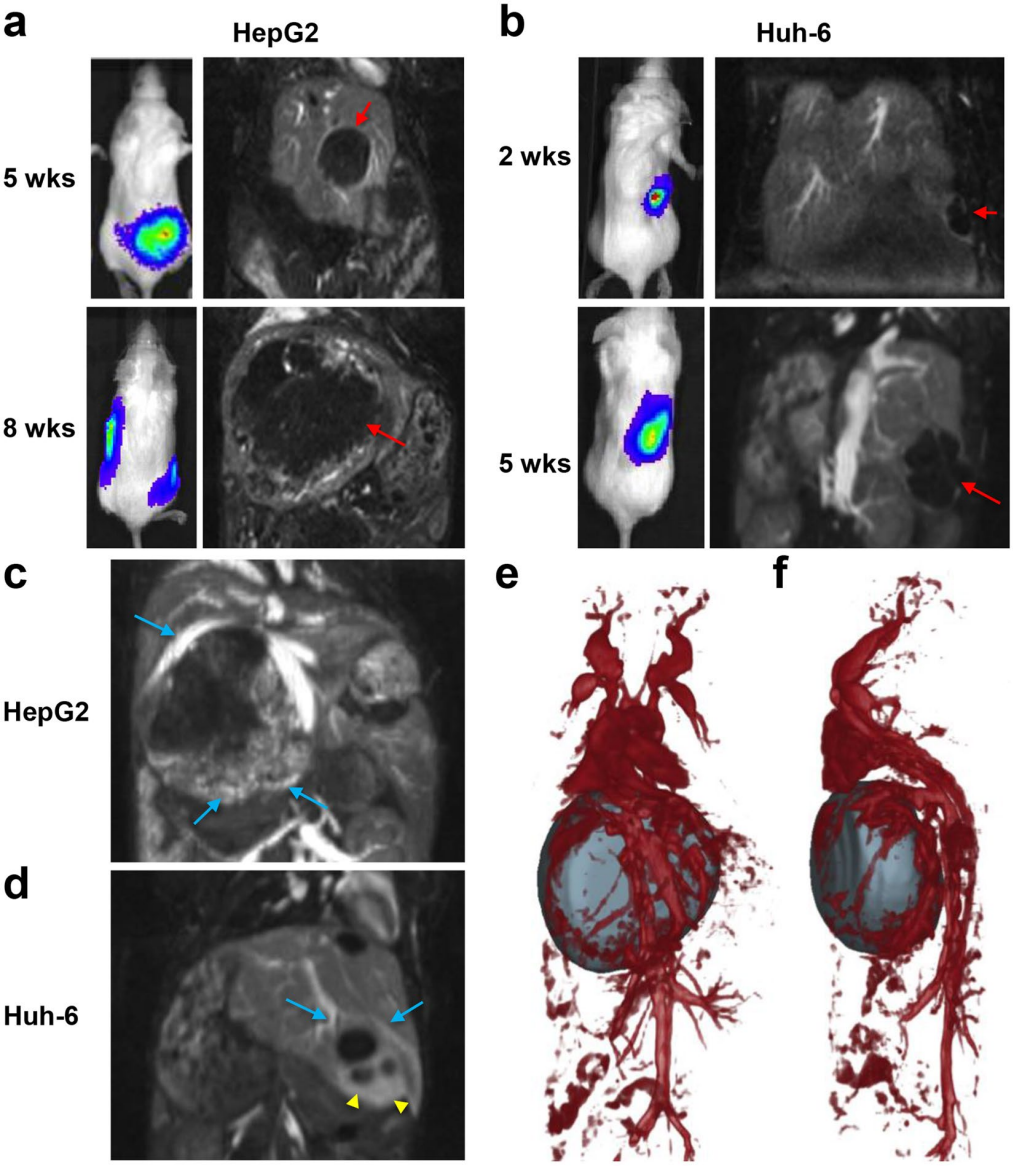
MRI of xenograft tumors and tumor-associated vasculature. (**a**,**b**) Representative contrast-enhanced T1-weighted coronal images showing the presence of tumor masses (red arrows) at early and late time points. Representative BLI images acquired on the same day are shown for comparison. (**c**,**d**) Representative contrast-enhanced T1-weighted coronal thick slab maximum intensity projection (MIP) abdominal images demonstrating the presence of increased vascularity (blue arrows) and highly permeable vessels (yellow arrowheads) in the peripheral regions of the tumors. (**e**,**f**) Coronal (**e**) and sagittal (**f**) 3D volume rendering MRI of HepG2 tumor and major hepatic blood vessels.

MRI was performed at early and late time points to confirm the presence of tumor and for 3D assessment of tumor burden. Intrahepatic tumors were visible as hypo-intense regions in the lobes of the liver (Fig. 3a,b). Contrast-enhanced T1-weighted imaging was performed using a long circulating blood pool liposomal contrast agent for assessing intra-tumoral vasculature and spatial relationships between tumor and major hepatic blood vessels. The blood pool agent showed the presence of intra-tumoral vasculature with a high degree of vascularity specificity at the tumor periphery (Fig. 3c–f). As the tumors grew, major hepatic vessels, including the inferior vena cava (IVC), were pushed from their regular anatomical orientation.

### Histological examination of xenograft tumors

**Figure 4:**
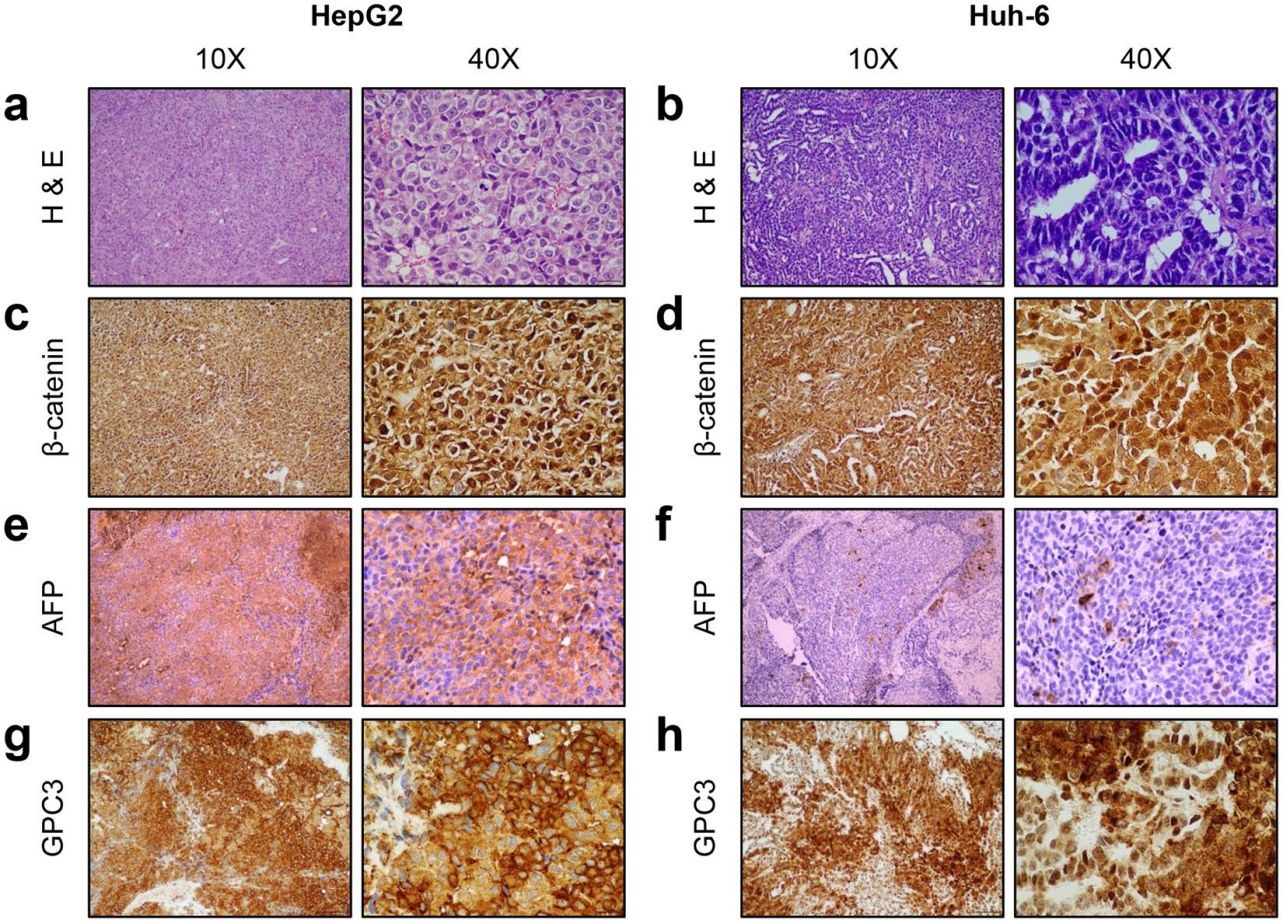
Histological examination of xenograft tumors. Samples representative of each xenograft tumor were stained with H&E (**a**,**b**) and antibodies recognizing β-catenin (**c**,**d**), AFP (**e**,**f**), and GPC3 (**g**,**h**), three proteins known to be elevated in HB. (**a**,**b**) HepG2 (**a**) shows classic HB embryonal morphology while Huh-6 (**b**) demonstrates areas of embryonal HB histology and a papillary pattern. (**c**,**d**) Cytoplasmic and nuclear β-catenin expression in HepG2 (**c**) and Huh-6 (**d**) samples. (**e**,**f**) AFP expression is elevated in HepG2 samples (**e**) but not in Huh-6 samples (**f**). (**g**,**h**) High GPC3 expression in HepG2 (**g**) and Huh-6 (**h**) tumors. Scale bars represent 100 μm in 10X images and 20 μm in 40X images (**a**–**d**,**g**,**h**) and 25 μm in 10X and 40X images (**e**,**f**).

At the conclusions of these studies, animals were euthanized and samples were harvested for histological and immunohistochemical analyses. Histological review of the xenografted tumors indicated that those generated with both HB cell lines resemble primary human HB samples to varying degrees (Fig. 4a,b). HepG2-derived tumors most closely resemble human HBs of homogeneous, embryonal phenotype (Fig. 4a). In contrast, Huh-6 tumors had morphologic characteristics that differ from the general histology of primary tumors (Fig. 4b). Huh-6 xenotransplants demonstrated an usual pattern with tumor cells organized in papillary structures generally uncharacteristic of HB (Fig. 4b). Of note, histology was similar to Huh-6 tumors obtained previously in the subcutaneous model^19^.

An immunohistochemistry panel, including AFP, β-catenin, and Glypican-3 (GPC3), commonly used in HB diagnosis and classification^20^ was employed to evaluate protein expression in the mouse xenograft tumors. AFP is variable in HB specimens since this protein is secreted and the key measurement is the levels of AFP in the serum^20,21^. Of the two cell line xenografts, only HepG2 tumors showed clear positive AFP staining throughout; Huh-6 tumors were predominantly negative with scattered positive patches of cells (Fig. 4e,f, Supplementary Fig. S1a–c). GPC3 is a reliable marker that is expressed in epithelial, fetal, and embryonal components and is negative in normal liver and benign tumor tissues^22^. Both HepG2 and Huh-6 xenograft tumors showed strong cytoplasmic and membrane staining for this marker (Fig. 4g,h). As a second assessment of expression of AFP and GPC3, we performed quantitative reverse transcription polymerase chain reaction (RT-PCR) (qPCR) experiments to measure levels of mRNA expression of these two HB markers in HepG2 and Huh-6 cells, in comparison to expression in the terminally differentiated hepatic cell line HepRG and in human fibroblasts (HFs). *AFP* and *GPC3* expression are both significantly elevated in HepG2 and Huh-6 cells (Supplementary Fig. S1d,e).

Finally, β-catenin is arguably the most important marker of HB as nuclear staining is used as a surrogate for the presence of mutations in the β-catenin gene, *CTNNB1*, which are commonly found in cases of HB^23^. Nuclear β-catenin expression is only seen in malignant hepatocytes^24^. In both the HepG2 and Huh-6 xenograft tumors, β-catenin staining was strong throughout the tumor nuclei (Fig. 4c,d). Taken together, histological analyses show that the xenograft tumors resemble primary HB tumors.

### Mutation analysis of cell lines and xenograft tumors

We further characterized the cell lines by performing mutational analyses of *CTNNB1* and the *Telomerase reverse transcriptase* (*TERT*) promoter gene previously reported to be present in each cell line (Table 1)^23^. Both cell lines carry mutations in *CTNNB1*. HepG2 carries a large in-frame deletion, p.W25_I140del (116 codons within exons 3 and 4)^25^, while Huh-6 carries a *CTNNB1* point mutation, p.G34V^25^. HepG2 is also reported to carry a mutation of the *TERT* promoter, G228A^26^.

**Table 1:** Mutation analysis of HB cell lines and xenograft samples.

	CTNNB1	TERT promoter
HepG2-C	p.W25_I140del (116 codons within exons 3 and 4)	G228A
HepG2-T	p.W25_I140del (116 codons within exons 3 and 4)	G228A
Huh-6-C	p.G34V	wild-type
Huh-6-T	p.G34V	wild-type

Two parental cell lines are HepG2-C and Huh-6-C; two cell lines grown *in vivo* as xenograft tumors are HepG2-T and Huh-6-T.

### Gene expression profiling of xenograft tumors

**Figure 5:**
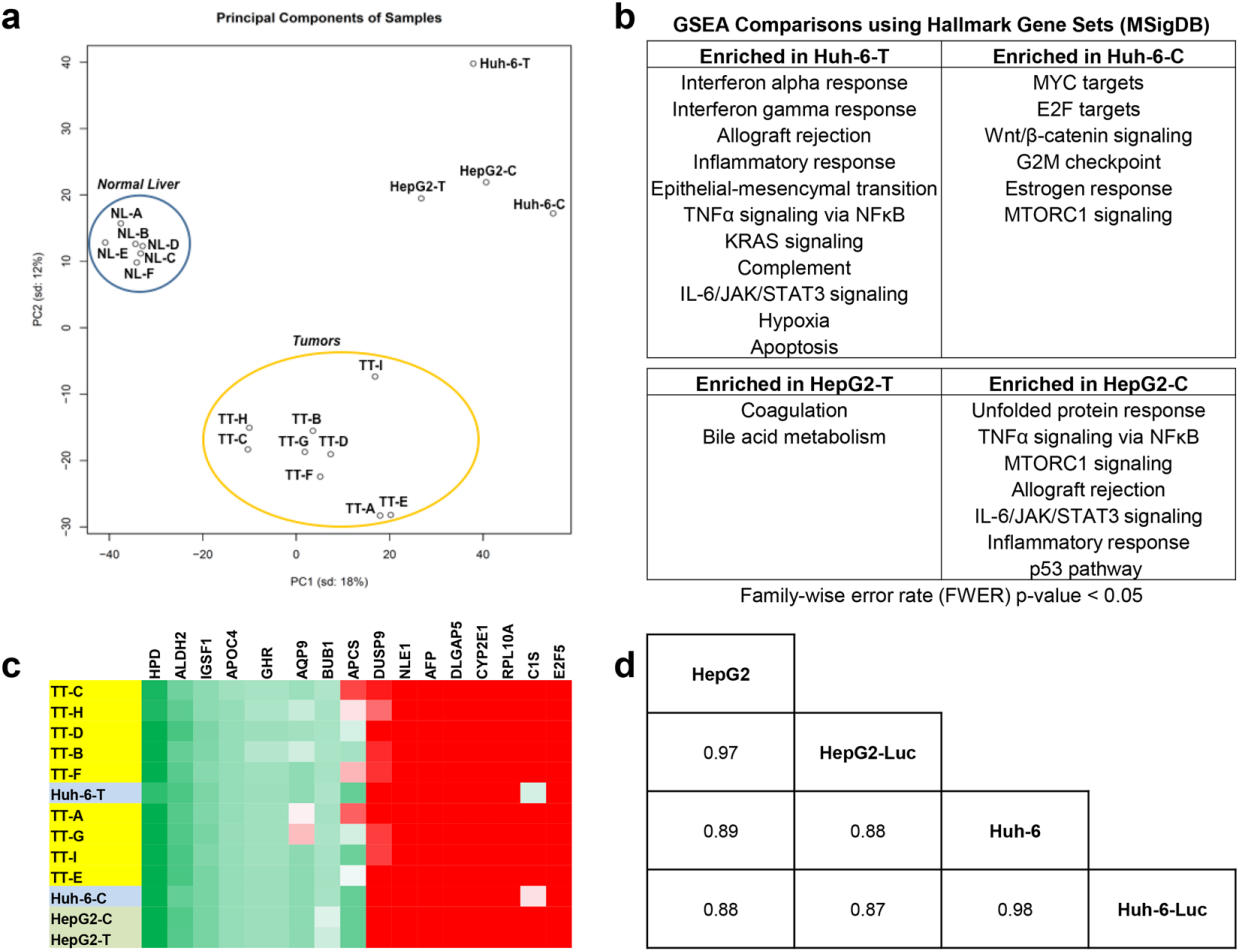
Transcriptome profiling of HB cell lines and tumor samples. (**a**) PCA of RNA sequencing data of two parental HB cell lines (HepG2-C, Huh-6-C) and two cell lines grown *in vivo* as xenograft tumors (HepG2-T, Huh-6-T), compared with RNA sequencing data of six normal liver samples and nine primary HB tumor samples. The two HepG2 samples and the Huh-6 sample grown *in vitro* form a cluster, indicating that their gene expression is similar to each other. The Huh-6 tumor sample is located far from the other clusters, showing that it has more transcriptome differences from the primary tumors and other cell line and xenograft samples. (**b**) GSEA to reveal transcriptome differences between cells grown *in vitro* and *in vivo* as xenograft tumors. (**c**) Molecular clustering of HB cell lines and tumor samples. Heat map analysis of 16-gene HB signature^27^ in nine primary HB tumors (yellow) and four HB cell line and xenograft samples (HepG2 (green), Huh-6 (blue)), normalized to six normal liver samples. The cell line and xenograft samples show the C2 signature, which is associated with a worse prognosis. (**d**) Spearman correlation of 18571 HUGO genes to indicate gene expression similarities of two parental cell lines and two *luciferase*-transduced cell lines all grown *in vitro*.

We used RNA sequencing to profile HepG2 and Huh-6 gene expression *in vitro*, including both parental and *luciferase*-transduced cell lines, and xenograft tumors generated *in vivo*. As an initial analysis, we compared transcriptome similarities between the *luciferase*-transduced cells and the parental cells (Fig. 5d) to show that the *luciferase-*transduced cells had almost identical gene expression as the parental cells. We then analyzed six normal liver samples, nine primary human untreated HB samples, and four *in vitro* and *in vivo* HepG2 and Huh-6 samples by principal component analysis (PCA). PCA of these profiles revealed three main clusters of samples: normal livers, primary tumors, and the *in vitro* and *in vivo* HepG2 and Huh-6 samples (Fig. 5a). *In vitro* and *in vivo* HepG2 profiles showed greater similarity than the corresponding Huh-6 profiles (Fig. 5a).

To reveal the major differences in the Huh-6 tumor sample that occur as a result of being grown *in vivo*, we conducted gene set enrichment analyses (GSEA) of each pair of *in vitro* cell and *in vivo* tumor samples with two gene sets from the Broad Molecular Signatures Database (Fig. 5b, Supplementary Table S2). Seventeen pathways in the Hallmark gene set were significantly changed between the two Huh-6 samples while nine pathways were significantly altered between the two HepG2 samples (Fig. 5b). Eleven pathways were significantly enriched in the Huh-6 tumor sample (Fig. 5b), including seven that are involved in the immune system and inflammation (interferon alpha response, interferon gamma response, allograft rejection, inflammatory response, Tumor necrosis factor alpha (TNFα) signaling via Nuclear factor kappa B (NFκB), complement, Interleukin 6 (IL-6)/Janus kinase (JAK)/Signal transducer and activator of transcription 3 (STAT3) signaling). These pathways were not enriched in the HepG2 tumor sample, although four were increased in the HepG2 cell sample (TNFα signaling via NFκB, allograft rejection, IL-6/JAK/STAT3 signaling, inflammatory response) (Fig. 5b). Enrichment for genes that function in the epithelial-mesenchymal transition, KRAS signaling, hypoxia, and apoptosis was also seen in the Huh-6 tumor sample (Fig. 5b), and these changes were not seen in either HepG2 sample. Unique differences seen in the Huh-6 cell sample included higher expression of MYC targets, E2F targets, Wnt/β-catenin signaling, G2M checkpoint, and estrogen response. Higher expression of Mammalian target of rapamycin complex 1 (MTORC1) signaling was seen in both the Huh-6 and HepG2 cell samples. Taken together, these statistical analyses suggest that more gene sets are changed between the Huh-6 *in vitro* and *in vivo* samples than the two HepG2 samples, many of which are connected to the immune response.

In a third, specific analysis of the RNA sequencing dataset, we analyzed the expression of a previously published prognostic 16-gene signature that has been reported to differentiate between a low risk, better prognosis HB cluster (C1) versus a high risk, poor prognosis HB cluster (C2)^27^. We used a heat map to classify expression in the two cell lines and corresponding xenograft tumor samples compared to the nine primary HB patient samples, all normalized to average expression in the normal liver samples (Fig. 5c). In general, all of the human HB tumor samples showed a C2 gene expression profile.

## Discussion

HB is the most common pediatric primary liver tumor. Although generally a rare cancer, it leads to the death of more than half of high-risk patients even with intensive chemotherapy and surgical interventions^3^. The current clinical trials in the U.S. (AHEP0731) and Europe (SIOPEL-4) all use non-targeted chemotherapy in their regimens including primarily cisplatin and doxorubicin^4^. These therapies have significant side-effects with about 50% of patients exhibiting ototoxicity and 5% showing cardiotoxicity^5,6^. Thus, novel therapeutic agents are needed. However, preclinical testing of such therapies has lagged due to the paucity of clinically relevant animal models of the disease. PDX models derived from primary tumors are very promising but have limited availability due to the rarity of the disease^14,28^. Subcutaneous and intrasplenic models utilizing commercially available cell lines do not accurately recapitulate tumors seen in patients^9,10^. In addition, a relevant study of intrahepatic xenograft tumors generated with HepG2 cells focused on this as a model of HCC instead of HB^13^. Hence, there is a compelling need for an intrahepatic xenograft model with human HB cell lines that replicates this complex disease, and, in this paper, we describe such a model that will enhance testing of new therapies.

Importantly, this intrahepatic HB xenograft model recapitulates the key hallmarks of the disease: elevated serum AFP levels, large exophytic tumors with active blood supplies, and embryonal histological phenotype with elevation of AFP, GPC3, and β-catenin. Although elevation of protein levels of AFP were not detected in Huh-6 tumors with immunohistochemistry experiments, significant increases in mRNA expression of both *AFP* and *GPC3* were seen in HepG2 and Huh-6 cells. Because of this discrepancy, we verified our data by staining the Huh-6 tumors with a total of four different AFP antibodies. With all four antibodies, the tissues were predominantly negative with limited scattered patches of positive cells. In the literature, it is still unclear whether detection of AFP with immunohistochemistry assays correlates with serum levels of this protein. In a large immunohistochemistry study of 83 patient samples comprising a mix of diagnostic tumor biopsies and post-chemotherapy, post-surgical specimens, no statistically significant correlation was found between levels of serum AFP at diagnosis and with expression of AFP in resected tumors^21^. We speculate that such secreted proteins have high rates of protein turnover in cells and thus are not always detectable with immunohistochemistry assays. This study shows that direct intrahepatic orthotopic injection of widely distributed HB cell lines in immunodeficient mice leads to primary hepatic tumors that morphologically mimic human HB tumors, and this is the first report that characterizes such cell line-derived xenograft tumors in relationship to primary HB tumors in order to demonstrate clear clinical relevance.

In our study, we examined the complete gene expression profiles of all cell lines and xenograft tumor samples in comparison to normal liver and HB tumor samples, which has not been described previously. In PCA of the total RNA sequencing dataset, both HepG2 samples and the Huh-6 sample grown *in vitro* clustered close to each other. However, the Huh-6 sample that was grown *in vivo* differed from what would be expected given its human HB tumor origin. Interestingly, gene expression changes in the Huh-6 tumor that seem to have occurred as a result of being grown *in vivo* result in cells with a profile different than the cell line grown *in vitro*. These results correlate with the histological review, as the Huh-6 xenograft tumor does not resemble primary HB disease as closely as the HepG2 tissue.

We speculate that this inconsistency may be due to the environment of the murine liver affecting the cell line as studies have shown that there are key differences between the human and mouse liver, especially in regards to transcriptional regulation and general gene expression among homologous genes^29,30^. Previous studies of *in vivo* models of HB have shown differences in histological appearance of cell line-derived tumors depending on their location of growth in the animal^9,10^. No previous studies have analyzed gene expression changes in HB cell lines grown *in vitro* and *in vivo*, but papers about other types of cancer have shown similar differences depending on whether cells are grown *in vitro* or *in vivo* and on location of growth *in vivo* ^31,32^.

In support of results from PCA, GSEA suggested more extensive divergence of the Huh-6 *in vivo* sample, including gene expression changes that indicate immune response and inflammation. It is well accepted that growth of human cells in animals requires the support of animal cells and that human and animal cells interface and interact with each other. Perhaps this contact is leading to unique upregulation of immune pathways within the Huh-6 cells, leading to more widespread alterations in gene expression in Huh-6 cells that are not seen in HepG2 tumors.

Taken together, the results of this study show that the commercially available HB cell lines can be used for *in vivo* preclinical testing; however, HepG2 more accurately mimics human HB. This study also makes it clear that more cell lines are needed to study HB biology and treatment response; therefore, large centers that treat many patients must come together to develop new patient-derived cell lines and xenografts. With the new international collaborations^33,34^ that have been formed to study HB, there will be more availability of tissues to develop these valuable resources.

## Materials and Methods

All animal procedures used in this study were performed under and in accordance with an animal protocol approved by the Institutional Care and Use Committee of Baylor College of Medicine.

### Cells and culture conditions

The HepG2, Huh-6, and HepRG cell lines used in this study were commercially acquired (HepG2: American Type Culture Collection (ATCC), Manassas, VA; Huh-6: Riken Cell Bank, Japan; HepRG: ATCC). Cell lines were grown at 37 °C in 5% CO_2_ in Eagle’s Minimum Essential Medium (EMEM, Lonza, Allendale, NJ) supplemented with 10% heat-inactivated fetal bovine serum (FBS, SAFC Biosciences, Lenexa, KS), 2 mM glutamine (Invitrogen, Carlsbad, CA), and 100 units/ml streptomycin/penicillin (Invitrogen). Cell lines were validated by DNA mutation analyses for known *CTNNB1* and *TERT* promoter mutations (see below).

### Orthotopic mouse model

*In vivo* studies were performed in female NOD/Shi-*scid*/IL-2Rγ^null^ (NOG) mice (Taconic Biosciences, Hudson, NY). 2 × 10^6^ HepG2 and Huh-6 cells transduced with *luciferase* and resuspended in 100 μl phosphate-buffered saline (PBS) were surgically implanted into either the right lobe of the liver through a right flank incision or the left lobe with a midline, abdominal incision. The mice underwent BLI beginning at 10 days after implantation and every week thereafter with the *In Vivo* Imaging System (IVIS, PerkinElmer, Waltham, MA), and luminescence flux was recorded to assess tumor growth. After seven weeks (HepG2) or four weeks (Huh-6), necropsy was performed, intrahepatic and extrahepatic sites of tumor were noted, and samples were harvested for immunohistochemistry and RNA isolation.

### ELISA to measure circulating AFP in mouse blood

Blood was drawn from the facial veins of mice harboring xenograft tumors at the indicated time points. AFP was measured in the serum of animals with an AFP ELISA kit (EIA-1468, DRG Instruments, Germany).

### *In vivo* MRI

MRI was performed on a 1.0 T permanent MRI scanner (M2 system, Aspect Technologies, Israel). A 35 mm volume coil was used for transmit and receive of radiofrequency (RF) signal. Mice were sedated using 3% isoflurane, setup on the MRI animal bed, and then maintained under anesthesia at 1–1.5% isoflurane delivered using a nose cone setup. Body temperature was maintained by circulating hot water through the MRI animal bed. Respiration rate was monitored using a pneumatically controlled pressure pad placed in the abdominal area underneath the animal. A long circulating liposomal-Gd blood pool contrast agent (SC-Gd liposomes) was systemically administered via the tail vein at a dose of 0.1 mmol Gd/kg and used for contrast-enhanced T1-weighted imaging^35^. High-resolution contrast-enhanced MRI (CE-MRI) was performed using a T1-weighted 3D gradient echo (GRE) sequence with the following scan parameters: echo time (TE) = 3.5 ms, repetition time (TR) = 20 ms, flip angle = 70, slice thickness = 0.3 mm, field of view = 54 mm, number of slices = 68, matrix = 180 × 180, NEX = 1, spatial resolution = 300 µm^2^, scan time ~5 min. Images were analyzed and processed in Osirix (version 5.8.5, 64-bit, Pixmeo, Bernex, Switzerland). Tumor volumes were segmented and 3D volume-rendered images were generated in Slicer (version 4.4.0)^36^.

### Immunohistochemistry of tumor tissues

Tissue samples were fixed in 4% paraformaldehyde (PFA, Alfa Aesar, Ward Hill, MA) overnight at 4 °C. Tissues were then dehydrated in 70% ethanol until processing in paraffin. Samples were processed in the Texas Medical Center Digestive Diseases Center (Houston, TX). H&E and immunohistochemical staining of AFP (CP028, Biocare Medical, Concord, CA (Supplementary Fig. S1a); AC-0166, Epitomics, Cell Marque, Rocklin, CA (Supplementary Fig. S1c); ab46799, Abcam, Cambridge, MA (Supplementary Fig. S1b); 180003, Thermo Fisher Scientific, Waltham, MA (Fig. 4e)), β-catenin (ab32573, Abcam), and GPC3 (GPC3, 11395, Santa Cruz Biotechnology, Dallas, TX) were performed. Imaging of tumor sections on slides was done on a DMi8 microscope (Leica, Germany).

### Quantitative RT-PCR with cell lines

RNA was extracted from cells with the Direct-zol RNA MiniPrep Kit (Zymo Research, Irvine, CA, USA). RNA purity and quantity were determined using a spectrophotometer measuring absorbance at 260/280 nm. cDNA was generated from total RNA with the SuperScript III First-Strand Synthesis System for RT-PCR (Invitrogen) or the qScript cDNA SuperMix (Quanta Biosciences, Gaithersburg, MD, USA). Taqman qPCR was done with TaqMan Universal Master Mix II (Applied Biosystems, Foster, CA, USA) and with the following primers (Applied Biosystems): *AFP* (Hs1040598_m1) and *GPC3* (Hs01018936_m1). *GAPDH* (Hs02758991_g1) was used as an internal control in all qPCR experiments. All experiments were run on a StepOnePlus Real-Time PCR System (Applied Biosystems).

### Mutation analysis of cell lines and xenograft tumors

DNA from frozen xenograft tissues and cell lines was extracted using the QIAamp DNA Mini Kit (Qiagen, Germany). Samples were treated with RNase A and eluted in Buffer AE. *CTNNB1* exon 3 and 4 and *TERT* promoter mutation status was determined by PCR of genomic DNA with primers listed in Supplementary Table S1 ^37^. Two-directional Sanger sequencing analysis of PCR products was completed with Mutation Surveyor v. 5.0.1 (Softgenetics).

### RNA sequencing of hepatic tumors and comparison with parental cell lines and liver cancer samples

RNA from frozen hepatic tumor samples was isolated using the *mirVana* miRNA isolation kit (Ambion, Austin, TX). Samples were treated with DNase 1 and eluted in nuclease-free water. RNA from xenograft tumors and parental cell lines was isolated using the RNeasy Plus Mini Kit (74134, Qiagen). RNA samples were submitted for RNA sequencing to the Baylor College of Medicine Genomic and RNA Profiling Core (Houston, TX) or to GENEWIZ (South Plainfield, NJ). RNA-seq FastQ files were processed using STAR^38^ and Cufflinks^39^, with alignment to Hg19/GRCh37. HB tumor and normal liver samples were processed via Illumuna HiSeq. 2500^40^. HepG2 was sequenced on both platforms, and a multiplicative scaling vector was used to scale the cell line and xenograft values for comparison with the patient tumor and normal liver samples. Fragments per kilobase per million (FPKM) values for 2324 genes were included after filtering for Human Genome Organization (HUGO) Gene Nomenclature Committee (HGNC) protein coding genes with average expression above 25 FPKM and coefficient of variation (CV) above 0.3 over the set of all samples. Spearman correlation of 18571 HUGO genes to indicate gene expression similarities of two parental cell lines and two *luciferase*-transduced cell lines all grown *in vitro*. PCA was implemented in R (Team, R. D. C. R: A language and environment for statistical computing, http://www.R-project.org (2014)) using the prcomp function with the scale parameter set to adjust variables to unit variance.

### GSEA of xenograft tumors and cell lines

Read FASTQ files were processed with RSEM (version 1.2.17)^41^ using the read aligner Bowtie2^42^ applied to the combined human and mouse NCBI Refseq (3/21/16) transcriptomes. Next, TPM values from human transcripts were selected and renormalized. Using a gene expression cutoff of 7.5 transcripts per million (TPM) and CV above 0.3 for HGNC protein coding genes, two sample comparison gene set enrichment was implemented using the GSEA (v2.23) program^43^ in pre-ranked mode with an extended “Signal2Noise” metric score for ranking. The Broad Molecular Signatures Database (MSigDB v5.2) sets h (Hallmark) and c2 (Curated gene sets) were used.

### Statistical analysis

The Kruskal-Wallis test was used to determine the statistical significance of *in vivo* tumor flux differences among the indicated time points.

### Data availability

The datasets generated during and/or analyzed during the current study are available from the corresponding author on reasonable request.

## Electronic supplementary material

Supplementary Information

Supplementary Table S2
