# Supplementary material for: A Novel Cell Line Based Orthotopic Xenograft Mouse Model That Recapitulates Human Hepatoblastoma
Source: Sci Rep. 2017 Dec 19;7:17751. doi: 10.1038/s41598-017-17665-8 (PMC5736579; doi:10.1038/s41598-017-17665-8)
Supplement: Supplementary file 1 — Supplementary Information [file 41598_2017_17665_MOESM1_ESM.pdf]

A Novel Cell Line Based Orthotopic Xenograft Mouse Model That Recapitulates Human Hepatoblastoma

Sarah E. Woodfield[1+], Yan Shi[1+], Roma H. Patel[1+], Jingling Jin[2], Angela Major[3], Stephen F. Sarabia[3], Zbigniew Starosolski[4], Barry Zorman[2], Siddharth S. Gupta[1], Zhenghu Chen[1], Aryana M. Ibarra[1], Karl-Dimiter Bissig[5], Ketan B. Ghaghada[4], Pavel Sumazin[2], Dolores López-Terrada[3], and Sanjeev A. Vasudevan[1*]

[1]Divisions of Pediatric Surgery and Surgical Research, Michael E. DeBakey Department of Surgery, Texas Children's Surgical Oncology Program, Texas Children's Liver Tumor Program, Dan L. Duncan Cancer Center, Baylor College of Medicine, Houston, TX 77030, USA.

[2]Department of Pediatrics, Dan L. Duncan Cancer Center, Baylor College of Medicine, Houston, TX 77030, USA.

[3]Department of Pathology and Immunology, Dan L. Duncan Cancer Center, Baylor College of Medicine, Houston, TX 77030, USA.

[4]Singleton Department of Pediatric Radiology, Texas Children's Hospital, Houston, TX 77030, USA.

[5]Center for Cell and Gene Therapy, Stem Cells and Regenerative Medicine Center, Department of Molecular and Cellular Biology, Dan L. Duncan Cancer Center, Graduate Program Department of Molecular and Cellular Biology, Program in Developmental Biology, and Program in Translational Biology and Molecular Medicine, Baylor College of Medicine, Houston, TX 77030 USA.

+These authors contributed equally to this work.

To whom correspondence should be addressed:

*Sanjeev A. Vasudevan, M.D., Divisions of Pediatric Surgery and Surgical Research, M.E. DeBakey Department of Surgery, Texas Children's Surgical Oncology Program, Texas Children's Liver Tumor Program, Dan L. Duncan Cancer Center, Baylor College of Medicine, Texas Children's Hospital, 1102 Bates Ave., Suite 460I, Houston, TX 77030-2399. Tel: 1-832-822-3135, Fax: 1-832-825-3141. Email: sanjeevv@bcm.edu.

Supplementary Information

Supplementary Figure S1. Expression of AFP and GPC3 in HepG2 and Huh-6 cells and xenograft tumors. (a-c) Huh-6 xenograft tumors are predominantly negative for AFP with limited scattered patches of positive cells. Scale bars represent 25 µm. (d,e) mRNA expression of *AFP* and *GPC3* is significantly elevated in HepG2 and Huh-6 cells. Error bars represent SD. Paired *t* test *P<0.05, **P<0.01, ***P<0.001.

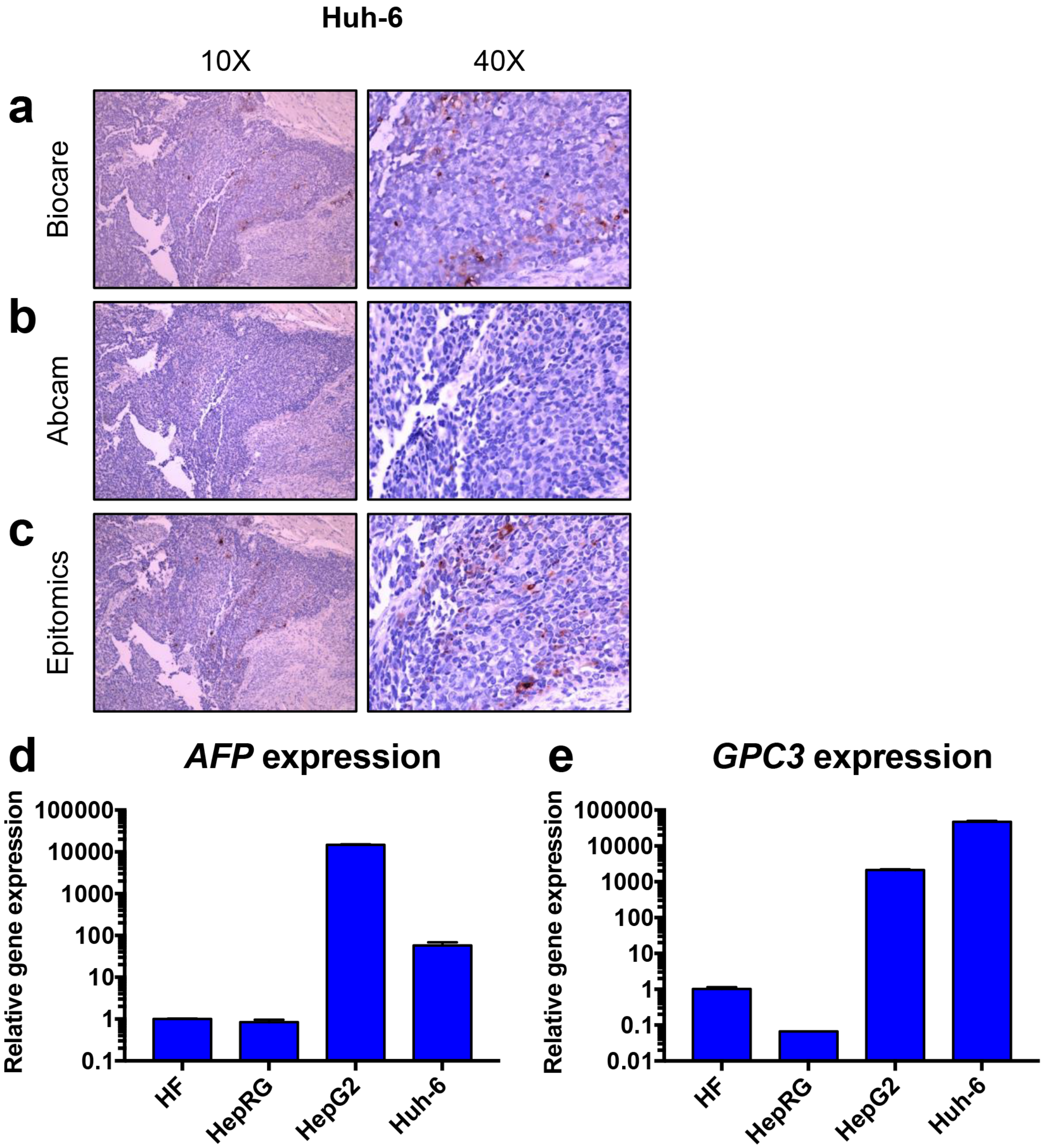
Huh-6
10X
40X
a
Biocare
b
Abcam
c
Epitomics
d
AFP expression
Relative gene expression
100000
10000
1000
100
10
1
0.1
HF
HepRG
HepG2
Huh-6
e
GPC3 expression
Relative gene expression
100000
10000
1000
100
10
1
0.1
0.01
HF
HepRG
HepG2
Huh-6

Supplementary Table S1. Primers for mutation analyses.

Gene	Primer ID	Sequence
CTNNB1	BCAT-F	5'-AGCGTGGACAATGGCTACTCAA-3'
CTNNB1	BCAT-R	5'-ACCTGGTCCTCGTCATTTAGCAGT-3'
TERT promoter	TERT-F	5’- TGTAAAACGACGGCCAGTAGTGGATTCGCGGGCACAGA-3’
TERT promoter	TERT-R	5' - CAGGAAACAGCTATGACCCAGCGCTGCCTGAAACTC - 3'

Supplementary Table S2. Curated gene sets enriched in cell lines grown *in vitro* and *in vivo* as xenograft tumors.
